# Supplementary material for: Hierarchical interactions between nucleolar and heterochromatin condensates are mediated by a dual-affinity protein
Source: Nat Cell Biol. 2025 Nov 24;27(12):2102–15. doi: 10.1038/s41556-025-01806-7 (PMC12717012; doi:10.1038/s41556-025-01806-7)
Supplement: Supplementary file 1 — Reporting Summary [file 41556_2025_1806_MOESM1_ESM.pdf]

nature portfolio

Corresponding author(s): Gary Karpen

Last updated by author(s): Oct 8, 2025

Reporting Summary

Nature Portfolio wishes to improve the reproducibility of the work that we publish. This form provides structure for consistency and transparency in reporting. For further information on Nature Portfolio policies, see our Editorial Policies and the Editorial Policy Checklist.

Statistics

For all statistical analyses, confirm that the following items are present in the figure legend, table legend, main text, or Methods section.

n/a	Confirmed	
☐	☒	The exact sample size (n) for each experimental group/condition, given as a discrete number and unit of measurement
☐	☒	A statement on whether measurements were taken from distinct samples or whether the same sample was measured repeatedly
☐	☒	The statistical test(s) used AND whether they are one- or two-sided<br>*Only common tests should be described solely by name; describe more complex techniques in the Methods section.*
☒	☐	A description of all covariates tested
☒	☐	A description of any assumptions or corrections, such as tests of normality and adjustment for multiple comparisons
☐	☒	A full description of the statistical parameters including central tendency (e.g. means) or other basic estimates (e.g. regression coefficient) AND variation (e.g. standard deviation) or associated estimates of uncertainty (e.g. confidence intervals)
☐	☒	For null hypothesis testing, the test statistic (e.g. F, t, r) with confidence intervals, effect sizes, degrees of freedom and P value noted<br>*Give P values as exact values whenever suitable.*
☒	☐	For Bayesian analysis, information on the choice of priors and Markov chain Monte Carlo settings
☒	☐	For hierarchical and complex designs, identification of the appropriate level for tests and full reporting of outcomes
☒	☐	Estimates of effect sizes (e.g. Cohen's d, Pearson's r), indicating how they were calculated

Our web collection on statistics for biologists contains articles on many of the points above.

Software and code

Policy information about availability of computer code

Data collection	ZEN Zeiss
Data analysis	ImageJ (Fiji), Arivis Vision 4D, GraphPad Prism, LAMMPS, Ovito, Github link: https://github.com/gauravbajpaimaths/Coarse-grained_model_of_nucleolar_heterochromatin_condensates

For manuscripts utilizing custom algorithms or software that are central to the research but not yet described in published literature, software must be made available to editors and reviewers. We strongly encourage code deposition in a community repository (e.g. GitHub). See the Nature Portfolio guidelines for submitting code & software for further information.

Data

Policy information about availability of data

All manuscripts must include a data availability statement. This statement should provide the following information, where applicable:

- Accession codes, unique identifiers, or web links for publicly available datasets
- A description of any restrictions on data availability
- For clinical datasets or third party data, please ensure that the statement adheres to our policy

All data supporting the findings of this study are available within the paper and its Supporting Information.

Research involving human participants, their data, or biological material

Policy information about studies with human participants or human data. See also policy information about sex, gender (identity/presentation), and sexual orientation and race, ethnicity and racism.

Reporting on sex and gender	n/a
Reporting on race, ethnicity, or other socially relevant groupings	n/a
Population characteristics	n/a
Recruitment	n/a
Ethics oversight	n/a

Note that full information on the approval of the study protocol must also be provided in the manuscript.

Field-specific reporting

Please select the one below that is the best fit for your research. If you are not sure, read the appropriate sections before making your selection.

☒ Life sciences ☐ Behavioural & social sciences ☐ Ecological, evolutionary & environmental sciences

For a reference copy of the document with all sections, see nature.com/documents/nr-reporting-summary-flat.pdf

Life sciences study design

All studies must disclose on these points even when the disclosure is negative.

Sample size	>30 nuclei were analyzed from at least 3 animals for each experimental group. In cell culture experiments, At least 10 cells were analyzed for each condition. While formal sample size calculations were not performed, 'n' was determined to be sufficient based on the reproducibility and consistency of the observed phenotypes among biological replicates. Precise n values for each experiment are reported in the manuscript.
Data exclusions	Microscopy data were only excluded from analysis when low fluorescence intensities caused inaccurate segmentation of the region of interest.
Replication	Experimental findings reported in the study were replicated at least once.
Randomization	Samples were allocated into experimental groups based on their genotype. Within each experimental group, samples were randomly selected for image acquisition and downstream analyses.
Blinding	Investigators were not blinded to group allocation during data collection or analysis. Experiments on control and test groups were performed concurrently, but they were processed in separate labeled tubes. To mitigate potential bias, all downstream measurements comparing different experimental groups were conducted using identical acquisition parameters.

Reporting for specific materials, systems and methods

We require information from authors about some types of materials, experimental systems and methods used in many studies. Here, indicate whether each material, system or method listed is relevant to your study. If you are not sure if a list item applies to your research, read the appropriate section before selecting a response.

Materials & experimental systems

n/a	Involved in the study	
☐	☒	Antibodies
☐	☒	Eukaryotic cell lines
☒	☐	Palaeontology and archaeology
☐	☒	Animals and other organisms
☒	☐	Clinical data
☒	☐	Dual use research of concern
☒	☐	Plants

Methods

n/a	Involved in the study	
☒	☐	ChIP-seq
☒	☐	Flow cytometry
☒	☐	MRI-based neuroimaging

Antibodies

Antibodies used	Antibodies used in this study: Rabbit anti-Fibrillarin (Abcam ab5821), Mouse anti-H3K9me2 (Abcam ab1220), Rabbit anti-H3K9me3 (Abcam ab8898), Mouse anti-Modulo (Gift from Mellone Lab), Mouse anti-Lamin, Dm0 (DSHB ADL67.10), Guinea Pig anti-Mxc (Gift from Duronio Lab), Rabbit anti-Pitchoune (Pit) was generated in this study by Pacific Immunology (CA), Goat-anti-Mouse Alexa Fluor 488 (Invitrogen A-11001), Goat-anti-Mouse Alexa Fluor 568 (Invitrogen A-11004), Goat-anti-Rabbit Alexa Fluor 488 (Invitrogen A-11034), Donkey-anti-Rabbit Alexa Fluor 568 (Invitrogen A-10042), Goat anti-Guinea Pig Alexa Fluor 647 (Invitrogen-A-21450), IgG (Invitrogen, 02-6102), Mouse anti-HP1a (DSHB, CA19)
Validation	Commercially purchased antibodies were validated by the suppliers. The antibody against Pit was generated for this study and validated by Western blotting in nuclear lysates, which detected a band at the predicted size of Pit. Additionally, when recombinant Pit was expressed in E. coli, the antibody detected it at the expected size.

Eukaryotic cell lines

Policy information about cell lines and Sex and Gender in Research

Cell line source(s)	S2R+ Drosophila cell lines were used in this study and procured from DGRC (Stock 150 ; https://dgrc.bio.indiana.edu//stock/150 ; RRID:CVCL_Z831). S2R+ are male Drosophila embryonic cells.
Authentication	The cell lines were not authenticated.
Mycoplasma contamination	Cells were not tested for mycoplasma contamination.
Commonly misidentified lines (See ICLAC register)	No commonly misidentified lines reported in the ICLAC register were used in this study.

Animals and other research organisms

Policy information about studies involving animals; ARRIVE guidelines recommended for reporting animal research, and Sex and Gender in Research

Laboratory animals	RFP-HP1a, GFP-HP1a (Karpen Laboratory); eGFP-Fibrillarin, RFP-Fibrillarin, eGFP-Mod, Pit-eGFP, eGFP-Nopp140, eGFP-Ns1 (shared by the Wieschaus laboratory), FM6/C(1)DX, y[*] f[1] (BDSC # 784), C(1)RM/C(1;Y)6,y[1]w[1]f[1]/0 (BDSC # 9460), Mat-alpha GAL4 (BDSC # 7063), Eyeless GAL4 (BDSC # 5534), Pit RNAi VAL20 (BDSC # 80368), Pit RNAi VAL22 (BDSC # 43984)
Wild animals	n/a
Reporting on sex	n/a
Field-collected samples	n/a
Ethics oversight	n/a

Note that full information on the approval of the study protocol must also be provided in the manuscript.

Plants

Seed stocks	n/a
Novel plant genotypes	n/a
Authentication	n/a
