# Supplementary figures and images for: Hierarchical interactions between nucleolar and heterochromatin condensates are mediated by a dual-affinity protein
Source: Nat Cell Biol. 2025 Nov 24;27(12):2102–15. doi: 10.1038/s41556-025-01806-7 (PMC12717012; doi:10.1038/s41556-025-01806-7)

**UNCROPPED WESTERN BLOT**  
related to  
**Fig. 5b**

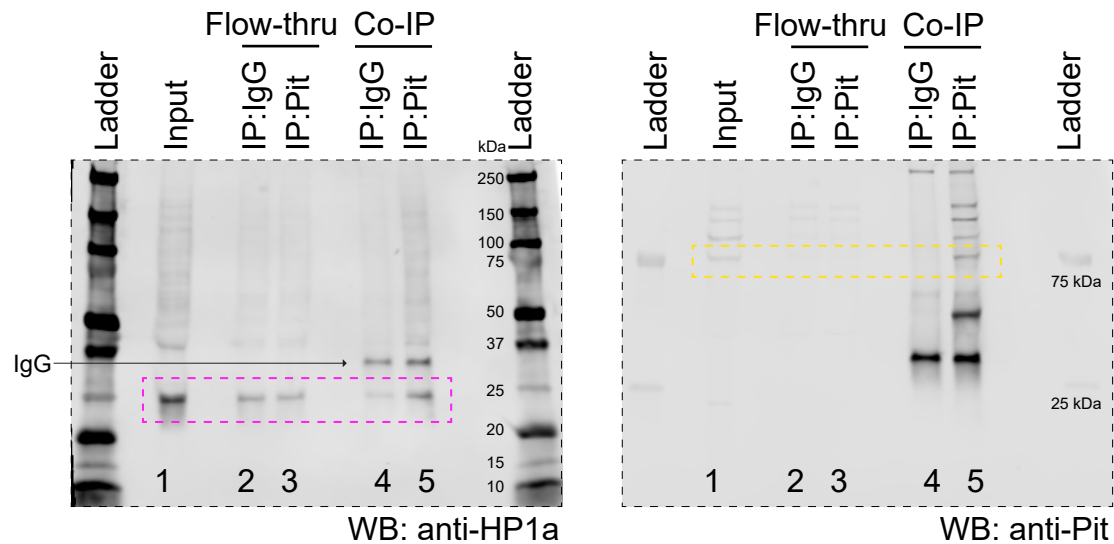

**UNCROPPED WESTERN BLOT**  
related to  
**Fig. 5d**

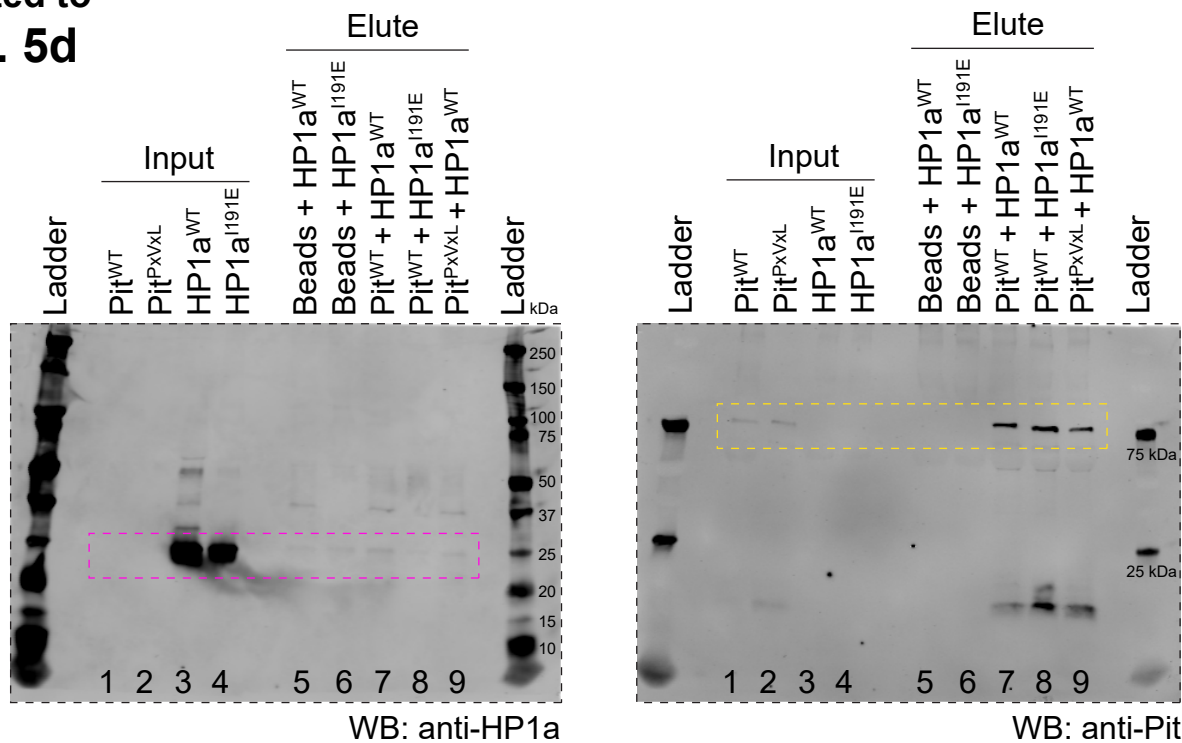

Supplement: Supplementary file 16 — Uncropped western blots. [file 41556_2025_1806_MOESM16_ESM.pdf]

# UNCROPPED WESTERN BLOT related to Extended Data Fig. 6c

**a**

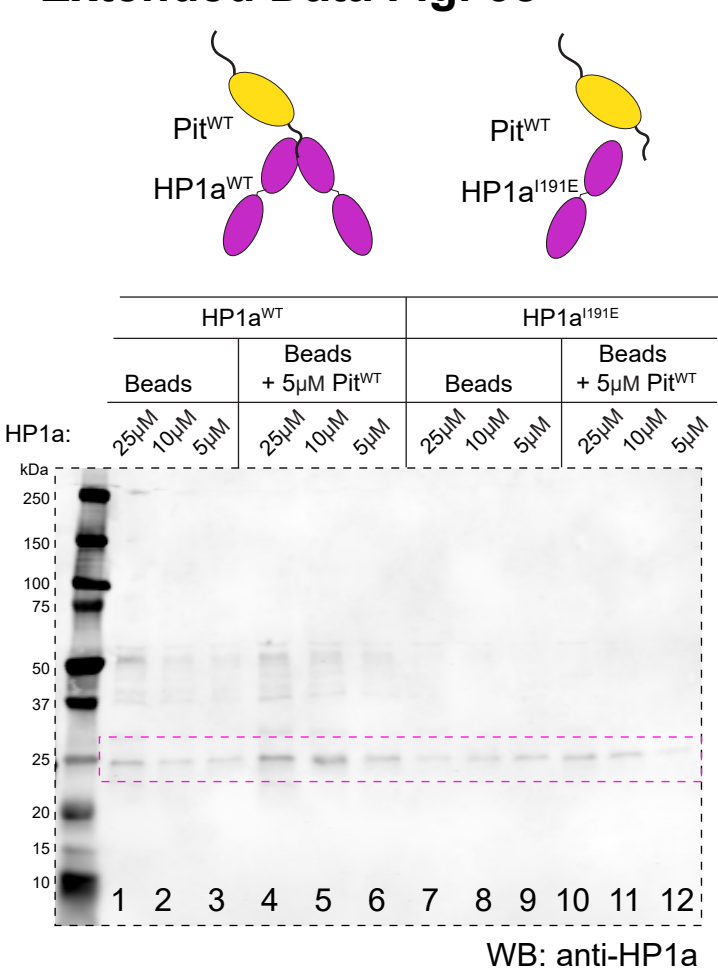

**b**

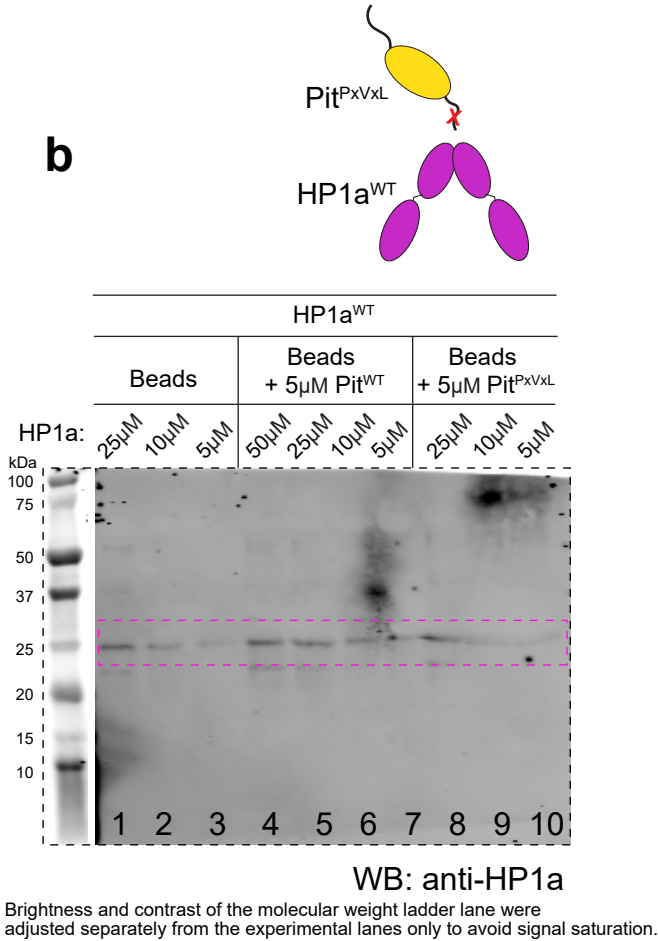

Supplement: Supplementary file 19 — Uncropped western blots. [file 41556_2025_1806_MOESM19_ESM.pdf]
